# Cross-reactivity and inclusivity analysis of CRISPR-based diagnostic assays of coronavirus SARS-CoV-2

**DOI:** 10.7717/peerj.12050

**Published:** 2021-10-01

**Authors:** Kashif Aziz Khan, Marc-Olivier Duceppe

**Affiliations:** 1 Department of Biology, York University, Toronto, ON, Canada; 2 Ottawa Laboratory Fallowfield, Canadian Food Inspection Agency, Ottawa, ON, Canada

**Keywords:** SARS-CoV-2, Sequence variation, Mutations, Diagnosis, CRISPR, COVID-19, Cross-reactivity, Cas9, Cas12, Cas13

## Abstract

Severe acute respiratory syndrome coronavirus 2 (SARS-CoV-2; initially named as 2019-nCoV) is the cause of the novel coronavirus disease 2019 (COVID-19) pandemic. Its diagnosis relies on the molecular detection of the viral RNA by polymerase chain reaction (PCR) while newer rapid CRISPR-based diagnostic tools are being developed. As molecular diagnostic assays rely on the detection of unique sequences of viral nucleic acid, the target regions must be common to all coronavirus SARS-CoV-2 circulating strains, yet unique to SARS-CoV-2 with no cross-reactivity with the genome of the host and other normal or pathogenic organisms potentially present in the patient samples. This stage 1 protocol proposes *in silico* cross-reactivity and inclusivity analysis of the recently developed CRISPR-based diagnostic assays. Cross-reactivity will be analyzed through comparison of target regions with the genome sequence of the human, seven coronaviruses and 21 other organisms. Inclusivity analysis will be performed through the verification of the sequence variability within the target regions using publicly available SARS-CoV-2 sequences from around the world. The absence of cross-reactivity and any mutations in target regions of the assay used would provide a higher degree of confidence in the CRISPR-based diagnostic tests being developed while the presence could help guide the assay development efforts. We believe that this study would provide potentially important information for clinicians, researchers, and decision-makers.

## Introduction

Severe acute respiratory syndrome coronavirus 2 (SARS-CoV-2; initially named as 2019-nCoV) was firstly isolated from a cluster of pneumonia patients in Wuhan, China and is the cause of novel coronavirus disease termed COVID-19 (Wu et al., 2020; Zhou et al., 2020; Zhu et al., 2020). The rapid spread of the virus has resulted in a declaration of a global pandemic by the World Health Organization (WHO) reaching more than 220 countries and territories (WHO, 2020c). SARS-CoV-2 has been classified as a member of the family *Coronaviridae* in the genus *Betacoronavirus* along with SARS-CoV and the Middle East respiratory syndrome (MERS)-CoV (Gorbalenya et al., 2020). The sequencing of the virus from patients early in the outbreak has shown that its single-stranded RNA genome is ~30 kb in size (Chan et al., 2020; Lu et al., 2020; Wu et al., 2020). The SARS-CoV-2 genome has been predicted to encode at least 10 open reading frames (ORFs) for structural and accessory proteins, based on similarity with SARS-CoV. As per current annotation (NC_045512.2), these viral ORFs encode replicase ORF1ab, spike (S), envelope (E), membrane (M), nucleocapsid (N), and at least six accessory proteins (3a, 6, 7a, 7b, 8, and 10) (NCBI, 2020). The pandemic has serious public health and economic implications. The day-to-day life of billions of people has been affected due to different forms of social distancing measures in place in different parts of the world to mitigate the spread of the virus. Thus, the widespread availability of rapid and reliable diagnostic testing is an important tool for policymakers to make public health decisions. The current diagnosis of COVID-19 relies on the molecular detection of viral RNA from patient samples using nucleic acid amplification tests (NAAT) like polymerase chain reaction (PCR) (WHO, 2020b). However, PCR requires specialized equipment and trained staff to perform the test and interpret the results, and thus is a challenge for remote low-resource settings. One of the alternatives being explored is the CRISPR-based nucleic-acid detection methods that may be particularly useful for screening outside the laboratory setting, for example at point-of-care, airports, offices and homes.

CRISPR-Cas (clustered regularly interspaced short palindromic repeats-CRISPR-associated), a component of the bacterial immune system to infectious nucleic acid, has been widely used as a gene-editing tool. This technology exploits the ability of Cas proteins to accurately target any region in DNA in association with CRISPR RNA (crRNA) that matches the target DNA with or without the requirement of a protospacer adjacent motif (PAM) (Moon et al., 2019). Initially explored for Cas9 protein (Pardee et al., 2016), the application of CRISPR in nucleic acid detection emerged as a viable tool with the discovery of promiscuous collateral cleavage activity of Cas12a (formerly Cpf1), Cas12b (formerly C2c1) and Cas13a (formerly C2c2) after target recognition (Chen et al., 2018; Gootenberg et al., 2017). Several CRISPR-based methods have been developed for the detection of RNA and DNA viruses (Jia et al., 2020; Strich, Chertow & Kraft, 2019). With the emergence of the novel coronavirus, scientists are rapidly employing these tools for the detection of SARS-CoV-2 from patient samples as an alternative to PCR. The Cas12a has been used for the diagnosis of COVID-19 from patient samples targeting viral genes N and E (Broughton et al., 2020). Similarly, Cas12b, Cas13a and FuCas9-based assays have also been developed for the detection of SARS-CoV-2 (Ackerman et al., 2020; Azhar et al., 2021; Guo et al., 2020). Cas12a-based DETECTR and Cas13a-based SHERLOCK have been approved by the US Food and Drug Administration (FDA) under Emergency Use Authorization (Mammoth, 2020; Sherlock, 2020) and FuCas9-based FELUDA has been approved for diagnosis of COVID-19 in India (Mitra, 2020). Several other CRISPR-based methods are also under development (Petrillo et al., 2020).

The molecular diagnosis of SARS-CoV-2 may be jeopardized by potential preanalytical and analytical vulnerabilities leading to false-positive or false-negative results (Lippi, Simundic & Plebani, 2020). As molecular diagnostic assays rely on the detection of unique sequences of viral nucleic acid, these are prone to mismatches due to genetic variability in the viral genome as well as cross-reactivity with the nucleic acid of other organisms present in the samples. The selectivity of an assay is generally validated in a laboratory using target strains, near-neighbour strains and other organisms. The use of bioinformatics tools and genome sequence databases can help to reduce wet-lab testing to a narrower focus and help to estimate more accurately the false-positive and false-negative rates of an assay (SantaLucia et al., 2020). It is known that mutations at primer/probe binding regions of the viral genome can result in potential mismatches and false-negative PCR diagnoses (Lefever et al., 2013; Stadhouders et al., 2010; Whiley & Sloots, 2005). We and others have concurrently demonstrated the genetic variability in the primer/probe binding regions of the SARS-CoV-2 genome highlighting the importance of periodic sequence verification for optimal virus detection (Farkas et al., 2020; Khan & Cheung, 2020; Osorio & Correia-Neves, 2020). Assay specificity remains a focus area in CRISPR-diagnostics as Cas proteins can result in a false-positive diagnosis due to their intrinsic capacity of mismatch tolerance. This risk has been minimized with the newer Cas proteins, Cas12a, Cas12b and Cas13, that have a lower tolerance for mismatches compared to front-runner Cas9 especially in the “seed” region (Safari et al., 2019). However, this raises the possibility that these tests may miss certain viral variants due to genetic variability in the regions targeted by these assays. The mismatch intolerant seed region of ~6 nucleotides is located in the PAM-proximal region for Cas12a (Chen et al., 2018; Kim et al., 2016) while the seed region is located in the central region of crRNA for Cas13a (Abudayyeh et al., 2017; Cox et al., 2017). *Francisella novicida* Cas9 (FnCas9) has been reported to have higher specificity and lower tolerance for mismatches compared to *Streptococcus pyogenes* Cas9 (SpCas9) tolerating only a single mismatch especially at the PAM-distal seed end (Acharya et al., 2019). It has been shown that even two mismatches would impede or even abolish the trans-cleavage activity of *Alicyclobacillus acidiphilus* Cas12b (AaCas12b/AapCas12b) (Teng et al., 2019). It is important to design crRNA adjacent to Cas-specific PAM that is common to all SARS-CoV-2 strains, yet unique to SARS-CoV-2 coronavirus with no cross-reactivity with the genome of the host and other normal or pathogenic organisms potentially present in the patient samples.

The objective of this study is the verification of the cross-reactivity and sequence variability within the target regions of CRISPR-based COVID-19 diagnostic assays, using publicly available sequence databases. The absence of any cross-reactivity and mutations in target regions of the assay used would provide a higher degree of confidence in the alternative tests being developed while the presence of mutations could help guide assay development efforts. We believe that this study would provide important information for clinicians, researchers and policy-makers.

## Methods

### CRISPR-based diagnostic assays and SARS-CoV-2 sequences

At least 15 crRNA of recently published CRISPR-based methods will be selected based on the literature review. The cross-reactivity and sequence variability within the target regions of CRISPR-based diagnostic assays will be determined using the protocol described below. The design planner is included in Table S1. The source code is available from the GitHub repository (https://github.com/duceppemo/CRISPR_Assay_Tester) and is easily installable with the Conda package manager. The script will be validated (and updated if necessary) as per the method described earlier (Khan & Cheung, 2020).

SARS-CoV-2 genome sequences deposited by laboratories around the world will be obtained from the NCBI virus database (Hatcher et al., 2017). A total of 400,000* near full-length sequences will be downloaded by applying the complete filter. The RNA genome of SARS-CoV-2 is shown in DNA format as per scientific convention. The complete genome of Wuhan-Hu-1 isolate, which is 29,903 bp long, will be included as a reference (NCBI accession number: NC_045512.2).

*The number of sequences in the NCBI database is growing on a daily basis and the exact number of included sequences would be updated in the 2^nd^ stage submission.

### Cross-reactivity analysis

Each crRNA sequence along with the PAM sequence, if applicable, (Table 1) will be analyzed for reactivity with the genome sequence of the human, seven coronaviruses and 21 other species including normal or pathogenic organisms that may potentially be present in patient samples. The complete list to be tested can be found in Table S2. This step will be performed using GGGenome nucleotide sequence search online server (http://gggenome.dbcls.jp/) (Naito et al., 2015) allowing several mismatches to check for ≥80% homology according to the requirement of WHO’s Emergency Use Listing for *in vitro* diagnostics detecting SARS-CoV-2 nucleic acid (WHO, 2020a). The potential hits on both orientations with different numbers of mismatches for each crRNA will be returned along with a summary of number of hits for different organisms. The hits with a match in the seed and the PAM region will be discussed.

**Table 1:** Cas proteins used in CRISPR diagnostics.

Cas variant	Enzyme	Organism	PAM
Cas12	LbCas12a (or LbaCas12a)	*Lachnospiraceae bacterium*	5′-TTTN
	AaCas12b (or AapCas12b)	*Alicyclobacillus acidiphilus*	5′-TTN
Cas13	LbuCas13a	*Leptotrichia buccalis*	Not applicable
	LwaCas13a	*Leptotrichia wadei*	Not applicable
Cas9	FnCas9	*Francisella novicida*	NGG-3′

### Inclusivity analysis

Multiple sequence alignment (MSA) of SARS-CoV-2 sequences will be performed using MAFFT (Multiple Alignment with Fast Fourier Transform) program v7.480 (Katoh & Standley, 2013) excluding low coverage sequences (>1% Ns) and using the Wuhan-Hu-1 genome (NC_045512.2) as reference. To evaluate the sequence variability in the target regions of each assay, referred here as the region of interest (ROI), the crRNA and PAM sequence will be extracted from all the entries in the MAFFT alignment file using the coordinates determined during cross-reactivity analysis. The MSA sequence for each ROI will be stratified by segregating sequences into discrete groups of identical sequence variants along with their frequency. To remove extremely low prevalent variants and sequencing errors in the data, only the sequence variants occurring in more than 0.01% of all sequences will be further considered by default. Sequences with ambiguous nucleotides and stretches of Ns in ROIs will be excluded from the analysis. The summary of mutated nucleotides for each target region will be returned and results will be reported as the frequency of hits with 100% match, hits with mismatches, and sequences below threshold. The frequency of sequence variants with mismatches in the seed and PAM region will be discussed.

## Supplemental Information

10.7717/peerj.12050/supp-1 Supplemental Information 1 Study design planner.

10.7717/peerj.12050/supp-2 Supplemental Information 2 List of Organisms to be tested for cross-reactivity.

## Additional Information and Declarations

### Data Availability

The following information was supplied regarding data availability: Python script is available at GitHub: https://github.com/duceppemo/CRISPR_Assay_Tester.
