# Supplementary material for: Cross-reactivity and inclusivity analysis of CRISPR-based diagnostic assays of coronavirus SARS-CoV-2
Source: PeerJ. 2021 Oct 1;9:e12050. doi: 10.7717/peerj.12050 (PMC8489407; doi:10.7717/peerj.12050)
Supplement: Supplemental Information 1 [file peerj-09-12050-s001.docx]

Table S1: Study design planner

| Question | Hypothesis | Sampling plan (e.g. power analysis) | Analysis Plan | Interpretation given different outcomes |
| --- | --- | --- | --- | --- |
| Is there any sequence variability and cross-reactivity in the SARS-CoV-2 genome within the target regions of CRISPR-based COVID-19 diagnostic assays? | - Potential match of crRNA+PAM with the genome of other organisms potentially present in the sample can lead to cross-reactivity.  - As the virus can potentially mutate during natural evolution, mutations in the target regions of CRISPR-based diagnostic assays can result in mismatches. | >400 000* viral isolates will be download from the NCBI virus database.  Inclusion criteria  Only complete (>29,000 bp) and high coverage (< 1% Ns) sequences  Exclusion Criteria  The sequences with stretches of Ns, and ambiguous sequences in the region of interest (ROI) will be considered low quality and will be excluded. | - Cross-reactivity will be analyzed using GGGenome  - For inclusivity analysis, sequences will be aligned using MAFFT and sequence variability in ROI will be traced using a python script.  - The complete genome of Wuhan-Hu-1 will act as a positive control (NCBI accession number: NC_045512.2). | - In the event of a negative result, it will be concluded that there is no evidence of a difference between crRNA+PAM and the genome of SARS-CoV-2/ other organisms tested.  - This will serve as a reference for clinicians and researchers using CRISPR-based assays for the detection of SARS-CoV-2. |
