# Supplementary material for: Cross-reactivity and inclusivity analysis of CRISPR-based diagnostic assays of coronavirus SARS-CoV-2
Source: PeerJ. 2021 Oct 1;9:e12050. doi: 10.7717/peerj.12050 (PMC8489407; doi:10.7717/peerj.12050)
Supplement: Supplemental Information 2 [file peerj-09-12050-s002.docx]

Table S2: List of Organisms to be tested for cross-reactivity

| **Host** | **Family *Coronaviridae*** | **Other high priority organisms** |
| --- | --- | --- |
| Human | SARS-CoV-2 | Adenovirus |
|  | Human coronavirus 229E | Human Metapneumovirus (hMPV) |
|  | Human coronavirus OC43 | Parainfluenza 1 - 4 |
|  | Human coronavirus HKU1 | Influenza A |
|  | Human coronavirus NL63 | Influenza B |
|  | SARS-coronavirus | Enterovirus |
|  | MERS-coronavirus | Respiratory Syncytial Virus |
|  |  | Rhinovirus |
|  |  | *Chlamydophila pneumoniae* |
|  |  | *Haemophilus influenzae* |
|  |  | *Legionella pneumophila* |
|  |  | *Mycobacterium tuberculosis* |
|  |  | *Streptococcus pneumoniae* |
|  |  | *Streptococcus pyogenes* |
|  |  | *Bordetella pertussis* |
|  |  | *Mycoplasma pneumoniae* |
|  |  | *Pneumocystis jirovecii* (PJP) |
|  |  | *Candida albicans* |
|  |  | *Pseudomonas aeruginosa* |
|  |  | *Staphylococcus epidermidis* |
|  |  | *Streptococcus salivarius* |
